# Epigenetic Reprogramming of TGF-β Signaling in Breast Cancer

**DOI:** 10.3390/cancers11050726

**Published:** 2019-05-24

**Authors:** Sudha Suriyamurthy, David Baker, Peter ten Dijke, Prasanna Vasudevan Iyengar

**Affiliations:** Department of Cell and Chemical Biology and Oncode Institute, Leiden University Medical Center, 2333 ZC Leiden, The Netherlands; s.suriyamurthy@lumc.nl (S.S.); d.a.baker@lumc.nl (D.B.); p.ten_dijke@lumc.nl (P.t.D.)

**Keywords:** breast cancer, epigenetics, epithelial to mesenchymal transition, signal transduction, SMAD, TGF-β

## Abstract

The Transforming Growth Factor-β (TGF-β) signaling pathway has a well-documented, context-dependent role in breast cancer development. In normal and premalignant cells, it acts as a tumor suppressor. By contrast, during the malignant phases of breast cancer progression, the TGF-β signaling pathway elicits tumor promoting effects particularly by driving the epithelial to mesenchymal transition (EMT), which enhances tumor cell migration, invasion and ultimately metastasis to distant organs. The molecular and cellular mechanisms that govern this dual capacity are being uncovered at multiple molecular levels. This review will focus on recent advances relating to how epigenetic changes such as acetylation and methylation control the outcome of TGF-β signaling and alter the fate of breast cancer cells. In addition, we will highlight how this knowledge can be further exploited to curb tumorigenesis by selective targeting of the TGF-β signaling pathway.

## 1. Introduction

**Figure 1:**
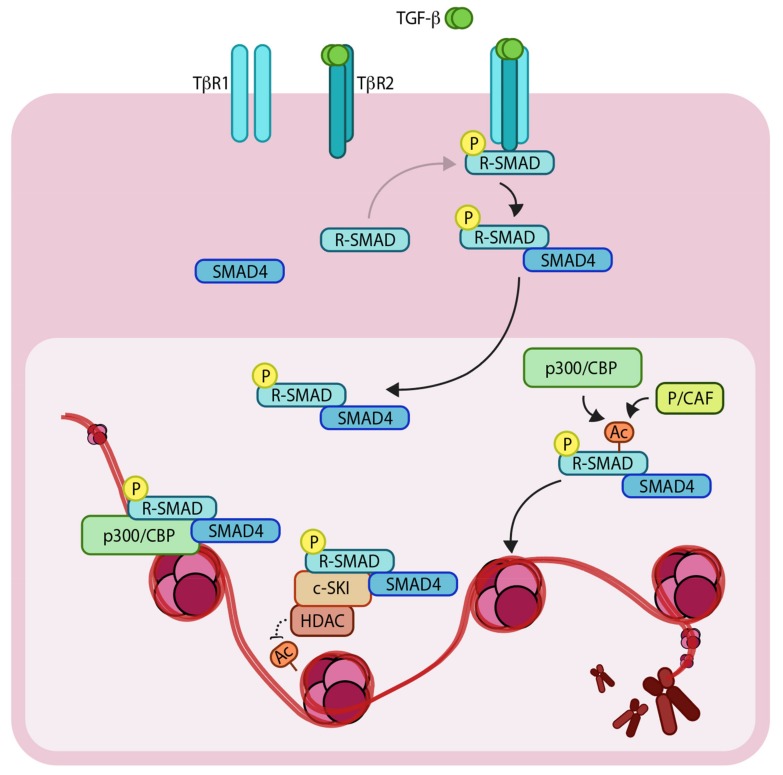
Schematic representation of the TGF-β (Transforming growth factor- β)/SMAD (SMA and MAD related protein)-induced transcriptional response mediated by coactivators and corepressors. The extracellular TGF-β signals via heteromeric complex of transmembrane TβR1 and TβR2 (TGF-β receptors 1 and 2). Upon TβR1 activation, R-SMADs (Regulatory SMADs) become phosphorylated and form heteromeric complexes with SMAD4. R-SMAD/SMAD4 complexes can act as transcription factors in concert with coactivators such as p300/CBP (CREB-binding protein) and p/CAF (p300/CBP associated factor), as well as corepressors such as c-SKI/HDAC (Histone deacetylase). ‘P’ in yellow circles indicates phosphorylation. Arrows denote addition of a modification or transfer of a protein complex and dotted arrow represents the reverse of this. ‘Ac’ indicates acetylation.

Transforming growth factor-β (TGF-β) is a multi-functional secreted cytokine that plays pivotal roles in early development and adult tissue maintenance [1,2]. Perturbation of TGF-β signaling has been implicated in a plethora of developmental disorders as well as diseases such as cancer. TGF-β exerts its cellular effects by binding to complexes of two related and ubiquitously expressed cell surface serine/threonine kinase receptors, i.e., TGF-β type 1 and type 2 receptor (TβR1 and TβR2, respectively [3,4]). Initially, TGF-β binds to TβR2, which in turn recruits TβR1 to form a heteromeric complex. The TβR2 phosphorylates and activates TβR1 [5], which is then capable of binding intracellular effector molecules, termed SMADs (SMA from ‘*Caenorhabditis elegans*, Sma gene’- and MAD from ‘Mothers against decapentaplegic’-related proteins). Upon ligand binding, the SMADs relay the signal from the cell surface to the nucleus resulting in changes in expression of specific target genes. The regulatory-(R) SMADs (i.e., SMAD2 and SMAD3) are phosphorylated directly by TβR1, which enables them to bind to a co-regulatory SMAD, called SMAD4 [6,7,8]. Thereafter, the R-SMAD-co-SMAD complex enters the nucleus and recruits additional co-transcriptional activators, repressors and/or co-factors (Figure 1). Notably, SMAD3 and SMAD4 (but not SMAD2) can bind directly to specific DNA motifs (5′-CAGA-3′ elements) to regulate expression of target genes. It’s also important to note that SMAD proteins alone bind to their consensus DNA binding sites with relatively low affinity but with significantly greater affinity in combination with other DNA binding transcription factors that are expressed in a cell-specific manner. This, in part, explains how specific transcriptional responses are generated in specific cells [9]. Interestingly, one of the direct target genes of the pathway is the inhibitory SMAD, *SMAD7*. SMAD7 acts as a negative regulator of TβR1 by recruiting SMURF2 (SMAD ubiquitination regulatory factor 2), an E3 ubiquitin ligase, to the receptor, which mediates its ubiquitylation and degradation, and thereby attenuating the signaling [10]. This negative feedback loop delimits the signaling response and thus prevents prolonged pathway activation.

TGF-β plays a complex, dual role in cancer progression: it behaves as a tumor-suppressor in normal and premalignant cells and as a tumor-enhancer during the more advanced stages of many cancers [11,12]. Consistent with its function as a tumor-suppressor, in many cancers, genes encoding components of the TGF-β signaling pathway have been either deleted or mutated. A classic example of this is the finding that *SMAD4* is frequently disrupted and inactivated in many types of cancer including 50% of pancreatic cancer patients [13]. However, this is not the case for breast cancer since such specific mutations in TGF-β signaling components are relatively rare. Such observations lead us and others to speculate that the fate of TGF-β signaling in breast cancer development is controlled by epigenetic mechanisms.

Dysfunctional epigenetic reprogramming of cells has been attributed to the development of a wide range of cancers [14]. Epigenetics describes biochemical changes to DNA or chromatin that alter the pattern of gene expression without modifying the actual DNA sequence, hence the Greek prefix “epi-” meaning (up)on/in addition to. The majority of epigenetic changes are mediated by histone modifying enzymes, regulators of DNA methylation and non-coding RNAs such as long non-coding (LncRNA) and microRNAs (miRNAs). To understand the nature of epigenetic change it is useful to first understand how DNA is packaged in cells. The average human cell contains approximately 2 meters of linear DNA which is stored in the nucleus as highly compact chromatin, consisting of repeating units, termed nucleosomes. Each nucleosome comprises short stretches of DNA, approximately 146 base pairs in length, wrapped around octamers of histone proteins (Figure 1).

### 1.1. Histone Modifications Govern Access of Transcription Factors to DNA 

The octamer consists of two copies of the “core” histone proteins, H3, H4, H2A and H2B. These histones can be changed biochemically by post-translational modifications (PTMs) enabling (or blocking) access of transcription factors to the promoters (or enhancers) of specific genes. Histones can undergo PTMs such as methylation of lysine and/or arginine residues, acetylation of lysine residues, ubiquitylation of lysine residues and phosphorylation of serine, threonine or tyrosine residues. Histones are methylated by histone methyltransferases (HMTs) [15], and the process can be reversed by histone demethylases. Collectively, these events can control expression of specific genes and play fundamental roles in many cellular processes [16]. Methylation of histones can lead to transcriptional repression or activation depending on the specific lysine or arginine modified. Histones can also be acetylated and deacetylated, which is mediated by histone acetyltransferases (or HATs) and histone deacetylases (or HDACs) respectively [17]. Acetylation of histones generally correlates with transcriptional activation as addition of the acetyl group lowers the positive charge on lysine residues and thereby reduces its affinity toward the negatively charged DNA, this promotes histone unwinding, allowing access of transcription factors to gene promoters. However, deacetylation of histones reverses this process. 

### 1.2. Epigenetic Regulators Modify TGF-β Signaling Components to Control the Genetic Output 

An intricate relationship between epigenetic regulators and TGF-β signaling has been established. As discussed below, many of the SMAD components are targets of such factors. The p300/CBP (CREB-binding protein) family of HATs were one of the first class of enzymes to be identified as co-activators of SMADs in the TGF-β signaling pathway [18]. Other HATs such as p300/CBP associated factor (p/CAF) and General control non-repressed protein 5 (GCN5) have also been shown to play important roles. The HATs, p300 and P/CAF, have been shown to acetylate SMAD2 and SMAD3 at specific lysine residues and thereby enhance their capacity to bind DNA [19]. An interplay between p300 and HDACs has also been shown to control the acetylation status of SMAD7 and regulate its stability [20]. Although, some of these modifications play an indirect role in the activity of proteins, there is clear evidence for direct effects exemplified by the fact that acetylation and ubiquitylation can occur at common lysine residues and the net effect of these opposing modifications determines the stability of the targeted protein. Moreover, a recent study showed that methylation of SMAD7 upon TGF-β stimulation regulates the ability of SMAD7 to bind TβR1 [21]. The epigenetic regulators c-SKI (C- for cytoplasmic and SKI represents Sloan-Kettering Institute, where it was first discovered) and SnoN (SKI-related novel protein N) were discovered as negative regulators of TGF-β signaling. c-SKI was first found to interact with SMAD2 and SMAD3, this binding competes with p300 and opposes acetylation [22]. Moreover, c-SKI also recruits HDACs, which act as transcriptional co-repressors limiting TGF-β target gene expression. A similar protein, SnoN, also limits TGF-β signaling in a similar fashion [23]. SnoN has also been shown to be a prognostic marker of estrogen receptor positive breast carcinomas [24].

The enzymes that underpin the so-called “histone code” (the totality of potential epigenetic marks that influence transcription), have been described as “writers, readers and erasers”. The enzymes that methylate or acetylate histones, such as HATs and HMTs are called writers. Enzymes that remove these modifications, such as HDACs, histone demethylases are called erasers. Enzymes that have either plant homeodomain (PHD), tudor-, chromo- or bromodomains, which recognize and bind to modified histone residues are described as the readers of the epigenetic information. A prominent example of a reader is TRIM33, which is also called Ectodermin; It belongs to the tripartite motif-containing (TRIM) E3 ubiquitin ligases. TRIM33 also has a PHD-bromodomain cassette that recognizes modified histones residues. The relationship between TRIM33 and TGF-β signaling has been explored in recent years and has been shown to play roles in development and tumor suppression by modulating SMAD activity in the nucleus [25,26].

### 1.3. Epigenetic Changes that Take Place at the Genomic DNA Level

**Figure 2:**
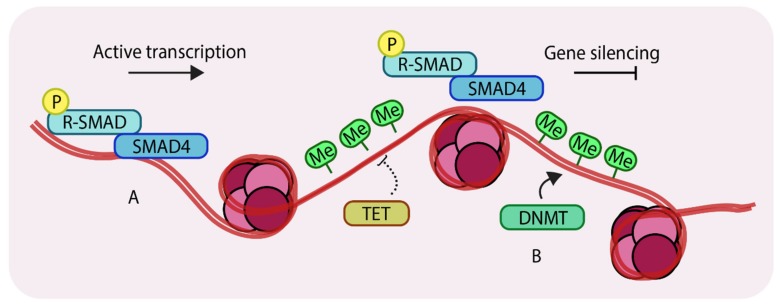
Methylation of genomic DNA. (**A**) unmethylated regions of DNA can allow binding of SMAD components and other transcription factors to enhance gene expression; (**B**) DNA methyltransferases (DNMTs) methylate genomic DNA, which inhibits binding of transcription factors thereby silencing certain genes. Ten-eleven translocation (TETs) antagonize DNMTs by removing methyl groups from DNA. ‘P’ in yellow circles indicates phosphorylation, ‘Me’ in green circles indicates methylation.

A different category of epigenetic change involves not histone modifications but direct modification of DNA. Certain regions of genomic DNA can be directly methylated by a class of enzymes called DNA methyltransferases or DNMTs. DNA methylation occurs through covalent modifications of cytosine sites in CpG dinucleotides. Such CpG dinucleotides occur in abundance in regions of DNA called CpG islands [27], which frequently characterize the promoters of actively transcribed genes. DNA methylation near transcription start sites (TSS) restricts the access of transcription factors to the promoter and thus acts to repress gene expression [28]. This process is counteracted by a family of enzymes called the Ten-eleven translocation (TET) enzymes [29]. Collectively, the relative actions of DNMTs and TETs determine the DNA methylation levels (Figure 2), and such DNA modifiers provide an extra layer of epigenetic control over gene expression.

Aberrant expression or dysregulation of the abovementioned epigenetic regulators can lead to activation of genes which are normally suppressed or vice versa. As will be discussed below, accumulating evidence points to the role for epigenetic regulators in driving the epithelial to mesenchymal transition (EMT) and hyperproliferation of breast cancer cells. EMT is a well-established process through which epithelial cells lose apical-basal polarity, tight junctions, and cell-to-cell contact whilst acquiring more mesenchymal features characterized by front-back polarity and increased expression of proteins such as vimentin and fibronectin [30]. This process occurs during early development as well as in normal adult tissue homeostasis such as during wound healing. Cancer cells harness this process to initiate migration, invasion and metastasis. Moreover, EMT has also been shown to contribute to cancer cell stemness and chemoresistance [31]. The remainder of this review will focus on how dysregulation of epigenetic factors controls the nature of TGF-β signaling-induced cellular responses and how these responses promote tumorigenesis. The first section will focus on patterns of DNA methylation that influence TGF-β signaling output by inducing cell-specific gene expression. The second section will consider recent advances in dissecting the roles of histone modifying enzymes that regulate the methylation and acetylation status of specific histones. Finally, we will discuss the emerging roles of non-coding RNAs in the epigenetic control of transcription. Although epigenetic modulators have been found to be dysregulated in many types of solid tumor, this review will mainly focus on the factors that function to promote breast cancer through TGF-β signaling pathway. Whilst it’s known that TGF-β signaling stimulates tumor progression through immune evasion, promotion of angiogenesis and the activation of cancer associated fibroblasts (CAFs), such effects on tumor stroma, which in part are also mediated by epigenetic changes, will not be discussed here. For this, the reader is referred to a number of excellent reviews [32,33,34,35,36].

## 2. Role of DNA Methylation in Breast Development and Tumorigenesis

The human genome encodes five DNMTs: DNMT1, DNMT2, DNMT3A, DNMT3B and DNMT3L. Coordinated changes in the global methylation status underlie normal development and alterations in the pattern of methylation have similarly been observed in various human cancers [37,38]. Mechanistically, there are a number of plausible consequences for changes in methylation, for example, increased methylation contributes to silencing of crucial tumor-suppressor genes. The following two recent developments describe genomic DNA methylation patterns that control the outcome of TGF-β signaling. Such modifications are observed during normal mammary development and also in the onset of breast cancer.

A striking illustration of this comes from studies of mammary gland development in higher (female) organisms. TGF-β signaling plays important roles in breast development especially during the changes accompanying puberty, pregnancy and lactation [39]. A number of studies have reported that TGF-β signaling inhibits mammary ductal growth in mice and also regulates the mammary epithelial stem cell populations [40,41]. Related to this, several reports also suggest that mammary epithelial stem cell populations undergo epigenetic changes during mammary gland development. One such study, using C57BL6 female mice, has demonstrated differences in genomic DNA methylation patterns in well-defined mammary stem cell lineages across different ages and reproductive stages [42]. Importantly, aging and reproductive stage could permanently alter DNA methylation profiles in some stem cell lineages. Interestingly, gene expression data established that TGF-β signaling is more active in certain stem cell populations in young (virgin) mice compared to mice of other stages (pregnant or older). TGF-β signaling pathway is known to be essential for stem cell function, by initiating expression of key genes such as *ID1*, *SNA1* and *FOXO* [43,44]. These studies highlight the roles of TGF-β signaling in the self-renewal and maintenance of specific stem cell populations and lend credence to the notion that aberrations in this pathway could give rise to premalignant cells [45]. Indeed, changes in the pattern of DNA methylation could be used as biomarkers to predict the early onset of breast cancer.

Several lines of evidence suggest that epigenetic changes underlie the tumor-suppressive or tumor-promoting effects of TGF-β signaling in breast cancer cells [46]. MDA-MB-231 is an aggressive breast cancer cell line whose metastatic potential is boosted by TGF-β signaling. Proliferation of the breast cancer cell line, HCC-1954, by contrast, is inhibited by TGF-β signaling [47]. SMAD3 chromatin immunoprecipitation and sequencing experiments (ChIP-seq) revealed that SMAD3 bound to open chromatin and sites without DNA methylation rather than regions with DNA methylation. This strongly suggests that expression of TGF-β target genes is dependent on patterns of DNA methylation which, in turn, can determine if TGF-β signaling acts as a tumor-suppressor or promoter. Notably, TGF-β target genes that were not expressed in HCC-1954 (but were expressed in MDA-MB-231) were characterized by higher levels of DNA methylation. By example, TGF-β induced the expression of the gene for *Limb Bud and Heart Development* (*LBH*), an essential gene that promotes stemness and inhibits differentiation, in MDA-MB-231 but not in HCC-1954, and this lack of induction was due to DNA methylation of the *LBH* promoter region in HCC-1954. Further analysis using loss-of-function studies of this gene in MDA-MB-231 revealed its importance in promoting tumor formation, whereas depletion of this gene (albeit lowly expressed) in HCC-1954 had no effect on the ability of TGF-β to inhibit proliferation of this cell line. These new results shed light on some of the epigenetic mechanisms that regulate TGF-β mediated gene expression in a cell-type specific manner. Future studies will establish if this is a general TGF-β signaling phenomenon. 

## 3. Methylation Status of Histones Govern TGF-β Mediated Changes

Methylation of histones at specific residues can lead to gene expression or repression depending on the residue and the nature of the modification. Specific lysine residues of histones can be either mono-, di- or tri-methylated. Arginine residues can be mono-, asymmetric di- or symmetric di-methylated. Each of these modifications have defined functions in the epigenetic program. Specific arginine and lysine residues of Histone 3 and Histone 4 play crucial roles in determining gene expression. For example, methylation at H3K9 (Histone 3 Lysine 9), H2K27, and H4K20 are associated with gene silencing whereas H3K4, H3K36, H3K79 are associated with active gene transcription [48]. It is important to note that these modifications are more complex than they appear. Differential degrees of modifications can be cues to specific signaling events. Mono-methylation of histones at specific lysine residues could have different biological consequences compared to di- or tri-methylation of the same residues. For example, H3K4 tri-methylation is often found associated with strongly expressed genes whereas H3K4 mono- and/or di-methylation is associated with low gene expression levels [49]. In many cases, mono-methylated lysine residues are primed for further rounds of methylation, which is mediated by specific HMTs. Such is the case with SETDB1 (Set domain bifurcated 1) which mediates H3K9 mono-methylation which is further acted upon by SUV39H1/2 (Suppressor of variegation 3–9 homolog 1/2) to induce H3K9 tri-methylation [50]. In the following section, we will highlight recent discoveries concerning the regulators of histone arginine and/or lysine methylation, and how these epigenetic marks influence TGF-β signaling output and breast cancer progression. 

### 3.1. PRMT5 Augments TGF-β-Mediated EMT

**Figure 3:**
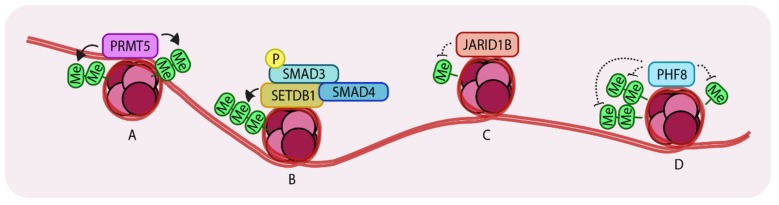
Representation of Histone methyltransferases and demethylases acting on histones. (**A**) PRMT5 (Protein arginine methyltransferase) (di-) methylates H3R2 (histone 3, arginine 2) and H4R3 which leads to enhanced transcription. (**B**) SETDB1 (Set domain bifurcated 1) tri-methylates H3K9 to repress *SNAI1* transcription. (**C**) JARID1B (Jumonji/ARID domain-containing protein 1B) de-methylates H3K4 to promote growth in breast cancer cells. (**D**) PHF8 (PHD finger protein 8) recognizes and demethylates H3K9me2, H3K29me2 and H4K20me leading to enhanced gene expression. Arrows indicate addition and dotted lines indicate removal of methyl groups. ‘Me’ in green circles indicates methylation.

Arginine methylation of histones is a well-characterized epigenetic modification. Such modifications are mainly (if not always) carried out by a family of enzymes called Protein arginine methyltransferases (PRMTs). Nine PRMT family members have been identified. These enzymes have various roles including transcriptional activation, signal transduction and ribosomal homeostasis [51]. Many PRMTs have been found to be overexpressed in different types of cancer. One family member, PRMT5, complexes with Methylosome protein 50 (MEP50) and promotes TGF-β-driven EMT and metastasis through histone 3 arginine 2 methylation-coupled transcription activation (H3R2me1/2) and histone 4 arginine 3 methylation-coupled transcriptional repression (H4R3me2). PRMT5 was found to be overexpressed in lung, breast and blood cancers and elevated expression also correlated with poor patient prognosis [52]. Interestingly, RNAseq transcriptional profiling of PRMT5 knockdown in A549 cells (a well-established lung cancer cell line model) showed that significant changes in the pattern of expression of genes are associated with the TGF-β pathway. Knockdown of this gene either genetically or pharmacologically (using the inhibitor, GSK591) caused a loss of colony formation ability and decreased the rates of proliferation, migration and invasiveness compared to control cells. At the molecular level, loss of PRMT5 leads to TGF-β signaling-dependent loss of E-cadherin and gain of expression of Vimentin and SNAIL, which are both normally induced by PRMT5 and are characteristic features of cells undergoing EMT (Figure 3A).

### 3.2. An Interplay between Acetylation and Methylation by SETDB1

It is known that TGF-β stimulation induces complex formation between R-SMADs, SMAD4 and p300/CBP (HATs) [18]. It has also been established that SMAD3 associates with SETDB1, a histone methyltransferase that (di- and tri-) methylates histone 3 at lysine 9, thereby repressing *SNAI1* transcription. The same site can also be acetylated (leading to transcriptional activation) suggesting that there is crosstalk between acetylation and methylation at histone 3 lysine 9, mediated by SMAD3/4, during EMT in breast cancer cells [53]. SNAIL is one of the “master” transcription factors that has a key role in inducing EMT [54]. TGF-β-mediated SMAD3 activation induces expression of *SNAI1* and also binds the encoded protein to repress *E-cadherin* expression. This also induces expression of mesenchymal markers in epithelial breast cancer cells. An additional layer of regulation has recently been unveiled revealing that SMAD3 recruits SETDB1 to the *SNAI1* promoter region, resulting in methylation of histone 3 lysine9 and repression of *SNAI1*. Interestingly, upon TGF-β stimulation, these cells lose expression of SETDB1 which leads to activation of the EMT program. In both NMuMG and HMLE breast cancer cell lines, TGF-β stimulation caused a decrease in the levels of SETDB1 as the cells gradually progressed from epithelial to mesenchymal states. Consistently, knockdown of this gene in NMuMG and HMLE cells enhanced the acquisition of mesenchymal characteristics after TGF-β stimulation, compared to control cells, which correlated with enhanced and prolonged expression of *SNAI1*. Loss of SETDB1 also enhanced mammosphere formation, anchorage-independent growth and gain of stem cell properties. In more mesenchymal cells such as MDA-MB-231, downregulation of SETDB1 led to decreased sensitivities to Camptothecin (an inhibitor of DNA topoisomerase 1) or doxorubicin. Loss of SETDB1 caused an increase in expression of the matrix metalloproteases, MMP-2 and MMP-9, which explained the enhanced invasiveness of these cells. Importantly, a positive correlation between breast cancer patient survival and expression of SETDB1 was noted. Mechanistically, it is possible that, in epithelial cells, this mechanism could act as a brake, which constrains EMT responses under basal levels of TGF-β (Figure 3B).

### 3.3. JARID1B Controls TGF-β-Mediated Growth Arrest

Histone methylation is a reversible process. Histone demethylation at specific lysine or arginine sites is carried out by Histone demethylases and generally, but not always, represses gene expression. A histone demethylase, JARID1B (Jumonji/ARID domain-containing protein 1B), which demethylates histone 3 lysine 4 (H3K4), was found to be overexpressed in luminal A and HER2 positive breast cancer subtypes due to increased gene copy number [55]. This gene is also highly expressed in ER+ (Estrogen receptor positive) luminal breast cancer cell lines e.g., MCF7 and T47D. Stable knockdown of JARID1B in these cell lines had significant growth inhibitory effects consistent with the notion that JARID1B is important for the proliferation of ER+ luminal breast cancer cells. Its knockdown in MCF7 cells resulted in a concomitant increase in SMAD2 phosphorylation. Gene expression analysis of the top 200 differentially expressed genes revealed upregulation of basal/stem cell genes and TGF-β target genes in JARID1B knockdown luminal cells compared to control cells. Notably, a TβR1 kinase inhibitor (LY2109761) rescued JARID1B knockdown-induced growth inhibitory effects suggesting that the inhibitory effects are TGF-β-dependent. In support of this view, downregulation of TGF-β signaling components like SMAD4 or TβR2 had similar effects. These observations suggest a novel role for a histone demethylase, JARID1B, specifically, that it is required to suppress the growth inhibitory effects of TGF-β signaling in luminal breast cancer cells. Loss of JARID1B in basal cell lines like SUM159PT and MDA-MB-231 decreased TGF-β pathway activity but did not modify cell growth, reinforcing the fact that TGF-β signaling output is controlled in a cell-type specific manner by histone modifying enzymes (Figure 3C).

### 3.4. A Subunit of the LSD1-CoREST Complex Controls the Expression of SNAIL

High mobility group domain containing protein 20B (HMG20B) is part of the Lysine-specific demethylase 1/REST co-repressor 1 (LSD1-CoREST) histone demethylase complex [56]. LSD1 has been shown to interact directly with SNAIL leading to gene repression of epithelial markers through demethylation of H3K4me1/2 [57]. Blocking this interaction suppresses motility and invasiveness of cancer cells [58]. A recent study revealed that the LSD1-CoREST complex is functionally different when HMG20A, a highly similar protein to HMG20B, is utilized instead of HMG20B [59]. The role played by HMG20A in the LSD1-CoREST complex was revealed using a retinal epithelial cell line as well as the breast cancer cell line models, NMuMG and MDA-MB-231. In NMuMG (control) cells, addition of TGF-β led to EMT, which was blocked in HMG20A knockdown cells. Further analysis showed that the HMG20A subunit is required for TGF-β-mediated repression of *E-cadherin*. Moreover, the levels of SNAIL were downregulated in HMG20A or LSD1 depleted cells, in support of the idea that HMG20A drives EMT as part of the LSD1-CoREST complex. In agreement with this, it was shown that the HMG20A subunit was essential for the cell migratory and invasive abilities of MDA-MB-231, as loss of expression of this protein abrogated the ability of the cells to invade through Boyden chambers. Such studies reveal the role played by HMG20A in the LSD1-CoREST complex, which is necessary for TGF-β-mediated EMT via epigenetic control of *SNAI1* expression.

### 3.5. KDM6B Stimulates SNAI1 Expression by Removing H3K27me3

The histone demethylase, KDM6B, activates gene expression by removing the repressive H3K27me3 mark from histones. KDM6B stimulates *SNAI1* expression during TGF-β-mediated EMT [60]. TGF-β stimulation has been demonstrated to induce early expression of KDM6B in NMuMG and HMLE cells and loss of KDM6B expression attenuated TGF-β mediated EMT. Correspondingly, RT-PCR profiling revealed that the induction of TGF-β-mediated *SNAI1* expression was suppressed in KDM6B knockdown cells compared to control. Furthermore, ChIP-seq analysis revealed that KDM6B promoted *SNAI1* expression by removing the inhibitory H3K27me3 histone mark. Interestingly, overexpression of KDM6B promoted an EMT-like phenotype in MCF10A by suppressing *E-cadherin* expression and inducing expression of mesenchymal markers. Given these findings, it was unsurprising that KDM6B was found to be expressed more highly in invasive breast carcinoma tissues compared to normal breast tissues. Accordingly, knockdown of KDM6B was found to significantly inhibit invasion in the highly metastatic breast cancer cell line, MDA-MB-231.

### 3.6. Demethylation by PHF8 Enhances EMT

The PHD finger protein 8 (PHF8) is a histone demethylase which acts on the H4K20me1, H3K9me1/2 and H3K27me2 marks to activate transcription of genes [61]. A significant body of evidence has highlighted a potential role for PHF8 in advanced stages of breast cancer [62,63]. Notably, PHF8 can induce malignant transformation of MCF10A in a 3D acinar formation assay. Overexpression of PHF8 in MCF10A cells led to an increase in the size of formed acini and a spindle-like morphology indicative of a mesenchymal phenotype. Consistently, PHF8 knockdown had the opposite effect. Overexpression of mutant PHF8 failed to produce comparable effects indicating that the demethylation activity of PHF8 is required for both malignant transformation and also for the induction of an EMT-like phenotype. It was shown that overexpression of PHF8 in these cells also led to enhanced appearance of stress fibers compared to control cells in response to TGF-β. Mechanistically, TGF-β induction recruits PHF8 to the TSS of *SNAI1* and suppresses the levels of H3K9me1/2, thus facilitating EMT (Figure 3D). A genome-wide expression analysis also confirmed that PHF8 overexpression led to upregulation of the EMT transcription factors SNAIL and ZEB1 (Zinc finger E-box binding homeobox 1). Data from the TCGA (The cancer genomic atlas) analysis revealed that PHF8 is upregulated in breast cancer malignancies, more specifically in invasive ductal and lobular breast carcinoma, invasive stroma and other rare types of breast carcinoma. Immunohistochemical analysis of clinical samples of breast cancer tissues also showed similar results.

## 4. An Interplay of Histone Acetylation and Deacetylation Regulates TGF-β Mediated Genetic Output

Acetylation of histones plays a crucial role in the epigenetic programming of genes. This modification can “open up” the chromatin to allow access of key transcription factors [64]. Given this, histone acetylation is most commonly seen near to and in the promoters of genes and enhancers. Histone acetyltransferases (HATs) catalyze the transfer of acetyl-CoA to the ε-NH_2_ group of lysine residues [65]. Common lysine residues acetylated by HATs include histone 3 lysine 9 (H3K9) and histone 3 lysine 27 (H3K27). HATs can be grouped into two main types: GNATs/GCN5 N acetyl-transferases and MYST (Morf, Ybf2, Sas2 and TIP60) acetyltransferases. The GNATs include HATs such as GCN5 or p/CAF. Other HATs such as p300 and CBP have intrinsic acetylase activity but lack HAT domains and are thus conventionally represented as an orphan class acetyltransferase enzyme [66]. Acetylation is reversible and deacetylation is executed by histone deacetylases (HDACs), which remove acetylation from histones leading to localized chromatin condensation. Some of the HDAC family members such as HDAC1 and HDAC6 have been previously shown to be overexpressed in breast tumors [67,68].

**Figure 4:**
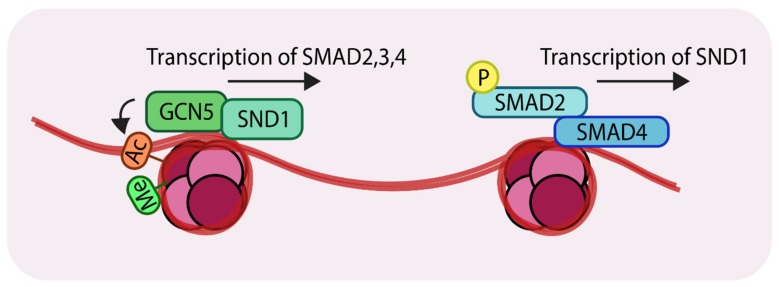
Representation of the action of the histone acetyltransferase, GCN5 (General control non-repressed protein 5) (*Left*); SND1 (Staphylococcal nuclease domain-containing 1) recruits GCN5 to the promoter regions of *SMAD2*, *3* and *4*. GCN5 acetylates at H3K9 which results in transcriptional activation. (*Right*) The SMAD complex can further enhance transcription of *SND1* creating a positive feedback loop. ‘Ac’ in orange circles indicates acetylation, ‘Me’ in green circles indicates methylation and ‘P’ in yellow circles indicates phosphorylation.

The transcription factor, staphylococcal nuclease domain-containing 1 (SND1), was recently found to recruit GCN5 to the promoter regions of *SMAD2*, *3* and *4* [69]. Comparing patient samples using immunohistochemistry identified higher expression of SND1 in metastatic breast cancer samples compared to non-metastatic tumors (without lymph nodes metastasis). Experiments in mice showed that MDA-MB-231 breast cancer cells spread to the lungs, whereas MDA-MB-231 cells lacking SND1 failed to efficiently form lung metastatic nodules in mice. Consistent with this finding, mice harboring knockdown cells had significantly longer survival times than those harboring control cells. Genome-wide ChIP-seq experiments revealed that SND1 targets genes involved in EMT and the TGF-β pathway. For example, these experiments revealed three SND1 binding sites in the promoters of *SMAD2*, *3* and *4*. To establish the biological relevance of these observations, overexpression of SND1 in MCF7 cells (with low endogenous SND1 levels) was shown to increase the mRNA levels (and protein levels) of SMAD2, 3 and 4. By contrast, knockdown of SND1, using shRNAs, in MDA-MB-231 (with higher endogenous SND1 levels) reduced mRNA levels (and protein levels) of SMAD2, 3 and 4. Such results demonstrate a direct relationship between the SND1 transcription factor and expression of key TGF-β signaling components. For transcription to proceed, acetylation of histone is necessary in order to open up the chromatin and expose the DNA binding motifs of transcription factors. Mechanistically, SND1 recruits a histone acetyltransferase GCN5 to the SMAD promoters in both MDA-MB-231 and MCF7. This promotes histone 3 lysine 9 (H3K9) acetylation. It was further established that H3K9Ac is coupled to H3K4Me3 [70], (Figure 4, left).

SND1 recognizes the H3K4Me3 modification on SMAD promoters and subsequently recruits GCN5 to these sites. Related studies revealed that TGF-β-mediated signaling can lead to *SND1* transcriptional activation [71], thereby forming a positive feedback loop to amplify TGF-β signals, which could account for the tumor-promoting properties of TGF-β signaling in advanced stages of metastatic breast cancer (Figure 4, right). In light of these observations, these studies have advocated the use of SND1 as a biomarker for advanced metastatic breast cancer.

## 5. Emerging Epigenetic Roles of Non-Coding RNAs

**Figure 5:**
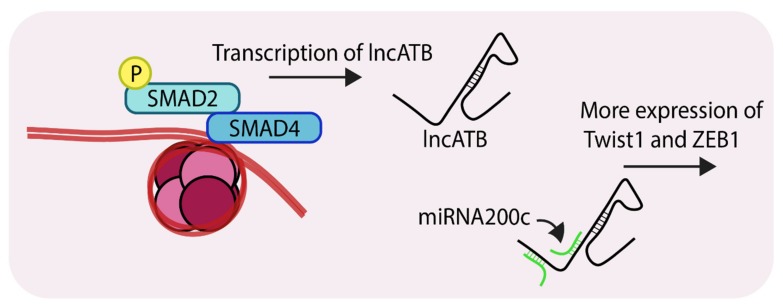
Representation of SMAD-mediated transcription of lncATB (Long non-coding RNA, activated by TGF-β). LncATB acts as sponge to soak up miRNA200c, which is a negative regulator of TWIST1 (Twist family bHLH transcription factor 1), this ultimately leads to its enhanced levels. ‘P’ in yellow circles indicates phosphorylation.

Non-coding RNAs are a group of molecules whose roles in cancer progression are beginning to emerge [72,73]. The long non-coding RNAs or lncRNAs form a sub-group of non-coding RNAs which are generally over 200 nucleotides in length [74]. LncRNAs such as HOTAIR, MALAT, H19 are correlated with breast tumor invasion, hyperproliferation and metastasis [75,76,77]. The lncRNA ATB (Activated by TGF-β) was first reported as a transcript which was overexpressed in hepatocellular carcinoma. Since then, recent reports have suggested it plays role in breast cancer progression, particularly during EMT and chemoresistance [78,79]. A recent publication described the mechanisms by which lncATB mediates EMT during breast cancer progression [79]. Compared to MCF10A cells, lncATB was expressed at higher levels in several triple-negative cell lines (MDA-MB-231, MDA-MB-436, BT-549 and BT-20). Analysis of MCF7 revealed that lncATB was stimulating TGF-β-mediated changes through the regulation of expression of EMT markers. Stable overexpression of lncATB in MCF7 caused upregulation of the mesenchymal markers ZEB1, TWIST1, N-cadherin and Vimentin, and down-regulation of E-cadherin. Importantly, expression of SNAIL and SNAIL2 were not significantly altered indicating that lncATB augments TGF-β-induced EMT by controlling the levels of ZEB1 and TWIST1. RNA pulldown experiments showed strong association between lncATB and miR200c, which is part of the microRNA 200 family which has an established role in EMT [80]. A search of the microRNA database revealed that TWIST1 harbors an miR-200c binding site in its 3’UTR (Figure 5). Overexpression of lncATB in MDA-MB-231, MCF-7 and BT-549 led to enhanced migration, invasion and clonogenicity. Mechanistically, lncATB acts as a sponge to soak up the miRNA200 family members and thereby upregulate TWIST1 levels. The expression of this lncRNA also correlates with patients exhibiting enhanced metastasis and poorer overall survival. 

SNAIL2, a closely related transcription factor to SNAIL, has also been implicated in the progression of metastatic breast cancer [81]; it is known to regulate the expression of the transcription factors ZEB1/2 [82]. Recent findings uncovered novel mechanisms through which TGF-β induces SNAI2 levels to promote EMT in breast cancer cells [83]. The study revealed an interplay between miR-203 (a member of the miR-200 family) and SNAI2 levels. Importantly, the results showed that miR-203 downregulates SNAI2 levels and, unsurprisingly, miR-203 was found to be down-regulated in metastatic breast cancer cells. 

## 6. Conclusions and Future Perspectives

The TGF-β pathway plays dual role in breast cancer: it is a tumor-suppressor in normal and premalignant cells and a tumor-promotor in advanced tumor stages. It’s important to note that these contrasting effects make it challenging to treat patients with inhibitors that target the TGF-β pathway. Related to this, TβR1 inhibitors have been shown to elicit side-effects in the cardiovascular system [84]. Nevertheless, to specifically curb the deleterious effects of the pathway, it is important to identify the factors responsible for controlling the molecular and cellular consequences of TGF-β signaling. Epigenetic mechanisms in the context of TGF-β signaling are clearly a critical dimension of the network that remains to be fully delineated. Several epigenetic regulators have been shown to directly control certain components of TGF-β signaling, which might plausibly offer a route to successfully targeting this pathway. Several research groups have studied HDAC and HAT inhibitors and novel inhibitors that target histone modifying enzymes are being developed and tested [85,86,87]. It is noteworthy that many of the recently developed inhibitors that target aberrantly expressed epigenetic regulators are being derived from naturally occurring substances such as Curcumin, Embelin, Garcinol and polyphenols in green tea. Recent studies have also highlighted a putative role of long non-coding RNAs in controlling the outcome of TGF-β signaling by modifying the epigenetic status of histones. In this light, antisense oligonucleotides (ASOs) are being developed as specific inhibitors of aberrantly expressed lncRNAs [88]. Further identification of tumor-suppressors and mechanisms of their epigenetic downregulation should be studied in future. Such results will shed light on novel epigenetic regulators that can be targeted which can enable re-expression of tumor-suppressor genes. Indeed, a clinical trial aimed to re-express estrogen receptors in triple-negative breast cancers was conducted (NIH clinical trial: NCT01194908). The trial used a combination of decitabine and panobinostat which is a non-selective HDAC inhibitor. Clinical trials have been conducted to deduce epigenetic alternations among patients as a method of stratification (NIH clinical trial: NCT01501656). These studies and developments raise the hope that the problems faced by targeting the TGF-β signaling pathway may be surmounted potentially yielding new therapeutic approaches for the treatment of breast cancer patients.

Recent developments have shed light on an emerging field of “Epitranscriptomics”, which has shown that mRNA molecules undergo post-transcriptional modifications. Accumulating evidence suggests that methylation of mRNA molecules is a critical form of its regulation. Studies have shown that mRNA molecules have the ability to get N6-methyladenosine (m6A) modifications, which are carried out by RNA methyltransferases. These mRNA modifications have been suggested to play critical roles in regulating its stability, degradation as well as translation. A recent study highlighted the role played by METTL14 (Methyltransferase like 14), a core component of RNA methyltransferase [89]. Knockdown of this protein resulted in reduced expression of several mRNA species such as TGF-β1, SMAD3, MMP9, VEGF (vascular endothelial growth factor) etc. in breast cancer cells. Furthermore, treating the METTL14 knockdown cells with TGF-β1 rescued the inhibitory effects. Another recent study described the interaction between RNA methyltransferase (METTL3-METTL14-WTAP, Wilm’s tumor-1 associated protein) complex with SMAD2 and SMAD3 [90]. The interaction was detected in human pluripotent stem cells, and this event led to rapid downregulation of specific SMAD2/3 transcriptional targets such as Nanog (the word Nanog is derived from Tìr nan Òg, the mythical Celtic land of youth). Such studies both highlight the importance of epitranscriptomics in TGF-β induced EMT and metastasis in breast cancer cells and also broaden our understanding as to how TGF-β signaling is reprogrammed in a cell-type specific manner. 

## Abbreviations

CBP	CREB-binding protein
ChIP-seq	Chromatin Immunoprecipitation-sequencing
c-SKI	Cytoplasmic-Sloan Kettering Institute
DNMT	DNA methyltransferase
GCN5	General control non-repressed protein 5
EMT	Epithelial to mesenchymal transition
ER+	Estrogen receptor positive
H3	Histone 3
H4	Histone 4
H3K4	Histone 3 lysine 4
H3K9	Histone 3 lysine 9
H3K4Me3	Histone 3 Lysine 4 tri-methylation
HAT	Histone acetyltransferase
HDAC	Histone deacetylase
HMG20	High mobility group domain containing protein 20
HMLE	human mammary epithelial cells
HOTAIR	HOX transcript antisense RNA
JARID1B	Jumonji/ARID domain-containing protein 1B
LBH	Limb bud and heart development
LncATB	Long non-coding RNA, Activated by TGF-β
LncRNA	Long non-coding RNA
LSD1-CoREST	Lysine-specific demethylase 1/REST co-repressor 1
MALAT	Metastasis associated lung adenocarcinoma transcript
MEP50	Methylosome protein 50
METTL14	Methyltransferase like 14
miRNA	microRNA
MMP9	Matrix metallopeptidase 9
Nanog	Derived from Tìr nan Òg, the mythical Celtic land of youth
p/CAF	p300/CBP associated factor
PHF8	PHD finger protein 8
PRMT	Protein arginine methyltransferase
SETDB1	Set domain bifurcated 1
SMAD	SMA and MAD related protein
SMURF2	SMAD ubiquitination regulatory factor 2
SNAI1	snail family transcriptional repressor 1
SND1	Staphylococcal nuclease domain-containing 1
SnoN	SKI-related novel protein N
SUV39H1/2	Suppressor of variegation 3-9 homolog 1/2
TCGA	The cancer genomic atlas
TET	Ten-eleven translocation
TGF-β	Transforming growth factor-β
TRIM33	Tripartite motif-containing 33
TSS	Transcription start site
TWIST1	Twist family bHLH transcription factor 1
TβR	TGF-β receptor
VEGF	Vascular endothelial growth factor
WTAP	Wilm’s tumor-1 associated protein
ZEB	Zinc finger E-box binding homeobox
